# Fall Detection Using Multiple Bioradars and Convolutional Neural Networks

**DOI:** 10.3390/s19245569

**Published:** 2019-12-17

**Authors:** Lesya Anishchenko, Andrey Zhuravlev, Margarita Chizh

**Affiliations:** Remote Sensing Laboratory, Bauman Moscow State Technical University, Moscow 105005, Russia; azhuravlev@rslab.ru (A.Z.); mchizh@rslab.ru (M.C.)

**Keywords:** bioradar, convolutional neural network, human fall detection, transfer learning, wavelet analysis

## Abstract

A lack of effective non-contact methods for automatic fall detection, which may result in the development of health and life-threatening conditions, is a great problem of modern medicine, and in particular, geriatrics. The purpose of the present work was to investigate the advantages of utilizing a multi-bioradar system in the accuracy of remote fall detection. The proposed concept combined usage of wavelet transform and deep learning to detect fall episodes. The continuous wavelet transform was used to get a time-frequency representation of the bio-radar signal and use it as input data for a pre-trained convolutional neural network AlexNet adapted to solve the problem of detecting falls. Processing of the experimental results showed that the designed multi-bioradar system can be used as a simple and view-independent approach implementing a non-contact fall detection method with an accuracy and F1-score of 99%.

## 1. Introduction

According to the UN [1], in 2019, 9% of the world’s population was aged 65 years or older; in Europe and Northern America, which have the most aged populations, the division was even more significant (18% of citizens were over 65) [1]. Every year the population over 65 is growing and by 2050 it will reach 15.9% for the World and 26.1% for Europe and Northern America regions, respectively [1]. It is a well-known phenomenon of global population aging, which is a result of increasing longevity and fertility decline.

The aging process is accompanied by negative changes in many systems and organs of the body, which may cause impaired coordination, loss of balance while changing the body position, a tendency towards fainting and dizziness, and others. These changes increase the risk of falls. According to the World Health Organization (WHO), ‘Approximately 28–35% of people aged 65 and over fall each year increasing to 32–42% for those over 70 years of age’ [2]. Falls frequently lead to functional dependencies. Moreover, falls are the second leading incident that cause accidental or unintentional injury deaths worldwide [2]. One of the factors influencing the severity of fall consequences in the elderly is the amount of time the victim remains immobile on the floor or ground waiting for help. The less time spent waiting for help, the less dreadful the consequences for health and more successful recovery and returning to the natural rhythm of life are.

Therefore, WHO claims fall-related research to be prioritized, while more and more scientists are paying their attention to the development of effective fall detection systems and methods.

There are many approaches and techniques that can be applied for fall detection. All of them may be divided into wearable and non-obtrusive methods. There are various wearable fall sensors currently on sale [3,4,5]. They utilize accelerometers [6] or gyroscopes [7] to measure acceleration changes in three axes or orientations in pitch, row, and yaw to detect falls. The main disadvantage of such devices is the high level of false-positive alarms, as well as the need to wear the device, which is often unacceptable to the end-user, as an elderly person may forget to put on this device or refuse to wear it for reasons of comfort.

Non-obtrusive non-wearable fall detection approaches seem to be more promising than wearable ones because they do not need any additional actions from the subject under observation after the fall detection system is installed at the user’s home. 

That is why during the last decade the scientific community has been actively developing new and improving existing non-obtrusive methods of automatic fall detection [8]. These methods can be video-based [9] or non-optical ones based on usage of depth cameras [10,11], ultrasonic [12], pressure [13], vibration [14], audio sensors [15], or Wi-Fi devices [16]. The main disadvantages for the non-obtrusive non-wearable fall detection methods at the moment are the problem of false alarms, high cost and privacy violation (in the case of using optical sensors). In addition, it should be noted that the video-based methods are sensitive to the lighting conditions of the room. Moreover, performing well under laboratory conditions, non-contact sensors have not been proved to provide the same performance in other surroundings and to be view-independent.

Some research groups proposed to used multi-sensor approaches which combine both contact and non-contact sensors to overcome the drawbacks of the previously mentioned methods [17,18]. However, such multi-sensors systems encounter more difficulties in implementing them in the field than systems utilizing only a single modality of sensor.

Fall detection is also possible by means of bioradiolocation [19]―the unobtrusive method for vital signs monitoring known since the 1970s [20,21]. It is based on the modulation of a microwave probing signal reflected from a human by the movement of a body’s surface, which may be caused by respiration, heartbeat, limb movements, etc. The main advantages of bioradiolocation are its non-contact and non-optical nature, thus it does not require any direct physical contact with the user and can sense even through optically opaque obstacles without any privacy violation.

It should be noted that the majority of papers dealing with fall detection by means of radars mainly present results obtained in laboratory conditions for similar positions of the falling subject and the radar during experiments [22,23,24], which makes such systems nonreliable if the position of the falling person has changed. Thus, the main disadvantage of the bioradar-based fall detectors is view dependency of the method performance.

This paper deals with the challenge that arises while applying the bioradar technique in realistic conditions, namely, a high impact of the falling subject orientation and distance toward the radar on the fall classification accuracy, which we propose to overcome by using a multi-radar approach. The novelty of the present work lies in the proposed architecture of the multi-bioradars system, which allows the observation of the subject from different angles, and the classification technique that makes the classification results reliable regardless of the position of the radar, overcoming the main disadvantage of the existing systems. 

## 2. Materials and Methods

### 2.1. Experimental Setup

**Figure 1:**
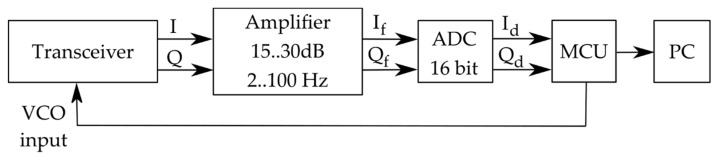
Scheme of the bioradar.

**Figure 2:**
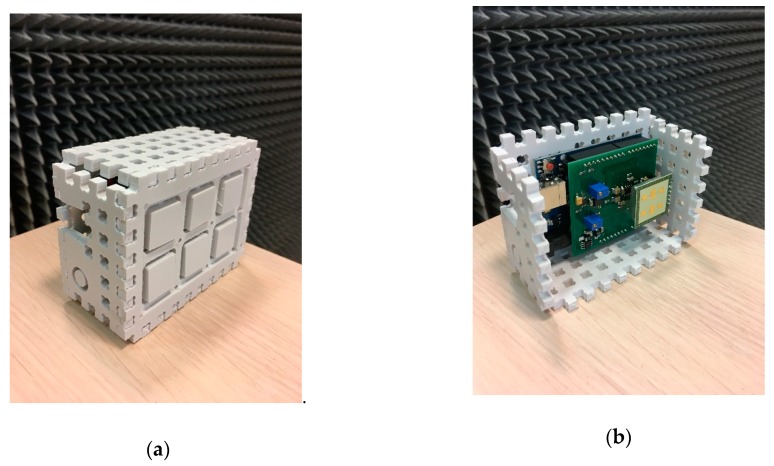
Bioradar prototype photos: (**a**) bioradar assembly; (**b**) housing panels removed.

The architecture of the bioradar used in the present work and the photo of the designed prototype are shown in Figure 1 and Figure 2, respectively.

The bioradar architecture is based on a concept of a low-cost portable bioradar proposed in our previous work [25]. The bioradar was designed by using a single-chip high sensitivity quadrature transceiver K-LC5 (RFbeam) [26], whose receiver has two separate output channels, i.e., the I (in-phase) and Q (quadrature) channels. This transceiver does not have an integrated amplifier, which makes it low-cost and suitable for the development of sensors in different arias of application. To make it suitable for a human fall detection task, we designed a customized amplifier adapting the scheme recommended by the manufacturer [26]. It limits the input signal bandwidth to around 1 to 100 Hz. The gain can be adjusted in the range of 15–30 dB, which allows detecting movements at the distance from 0.5 to 6.0 m between the bioradar and the subject.

Two filtered and amplified quadratures (If and Qf) are put through a 16-bit analog-to-digital converter (ADC) ADS1115 with a sampling rate of 250 sps for each channel. We used an Arduino UNO board as a microcontroller unit (MCU) to send the digitalized quadratures (Id and Qd) through a serial port to the personal computer (PC) for further processing. Furthermore, MCU was used to adjust the probing frequency of the bioradar by setting the level of the transceiver VCO input according to Table 1. It is needed to prevent interference between the probing signals of bioradars used simultaneously. According to the data reported in Table 1, the maximum power density radiated by the radar is less than 3 µW/cm^2^. Such a value satisfies the Russian safety standard for microwave emission, which is 25 µW/cm^2^ in the frequency range 3–300 GHz (for 24 h exposure).

**Figure 3:**
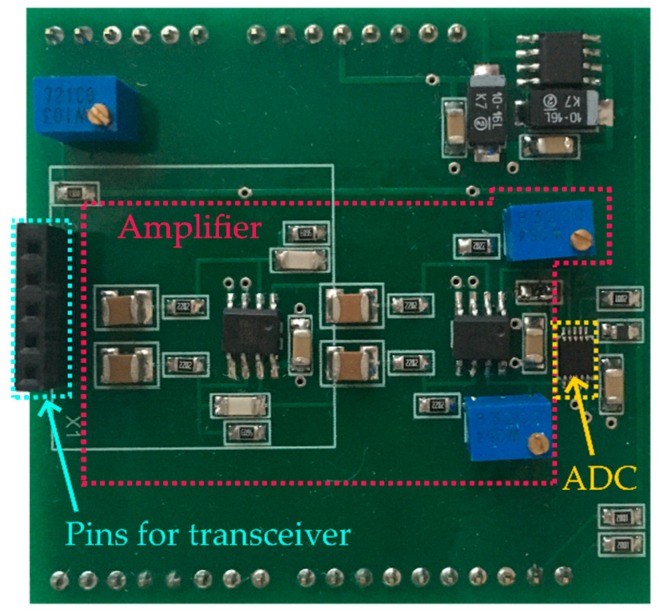
Designed shield for Arduino UNO board.

As it can be seen in Figure 3, the customized amplifier and ADC were placed on a shield for the Arduino UNO board. The K-LC5 transceiver was plugged into the shield through pins.

### 2.2. Description of the Experimental Procedure

The experiments were conducted to investigate the possibility of bioradars to detect fall events performed on different radar distances and angles. Experiments were carried out from June to July 2019 with the participation of five healthy adults (two males and three females) in the age group between 22 and 41 years. All subjects provided written informed consent prior to the start of the experiments. For the experiments involving human participants, an ethical approval was obtained on 1 March 2018 from the ethics committee of BMSTU. Information about the volunteers is given in Table 2.

**Figure 4:**
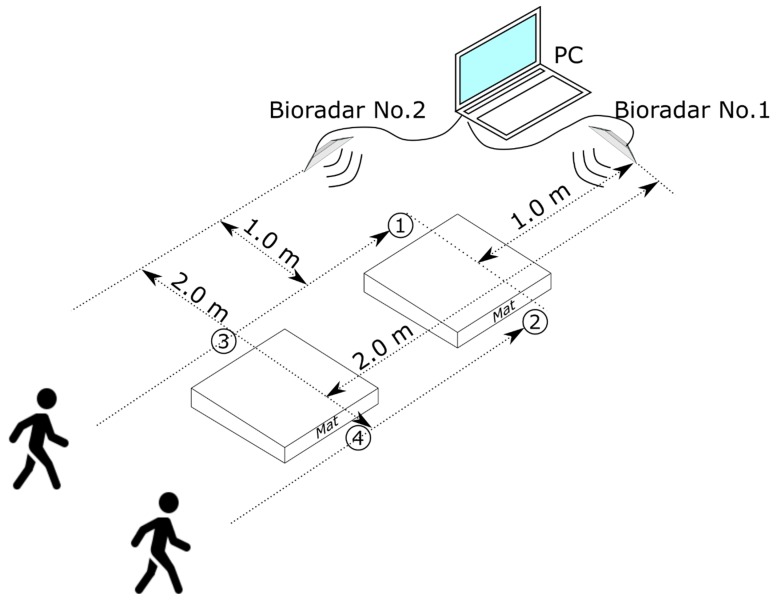
Scheme of the bioradar experiment.

The scheme of the experiment is shown in Figure 4.

The activities and falls were recorded in the furnished living room of 6.0 × 3.5 m size with the ceiling of 2.8 m height by two bioradars located at an angle of 90 degrees to each other. Such mutual positioning of bioradars allows observing the examinee from different viewing angles and guarantees that even if the subject’s movement is partially blocked by his/her body for one of the bioradars, this movement pattern will be observed by the other one. The bioradars were located at 80 cm above the floor: One for a frontal view and the second for a lateral view with respect to the volunteer.

Unlike other works in which falls were performed at the same place of the experimental scene [18,22,23,24], in the present work we try to solve a more challenging task, namely to detect falls performed at different positions. To do so, the volunteer was asked to enter the room and imitate falling or normal daily activity while reaching one of four points (points 1–4 in Figure 4), thus the range between the subject and the radars varied from 1.0 to 2.0 m, moreover, for points 3 and 4 in Figure 4, the volunteer was only partially observed by bioradar No. 2.

During the experiment, the subject was asked to perform various types of daily physical activity (entering and exiting the premises, doing sport exercises and housework, lying down on the mat and getting up from it), as well as to simulate falls of two types: slipping and loss of consciousness. Both types of falls were performed with different orientations (backward, forward, right and left side). The duration of each bioradar record was 10 s. In total, 350 bioradar records were made, including 175 with one fall episode and 175 with daily activities. Each of them was marked as ‘fall’ if it contained a fall episode, or ‘not fall’ if not. The starting time of fall events varied from record to record to make it equally distributed along the 10 s record duration. Such even distribution of fall events in time helped better generalization of the classification algorithm.

**Figure 5:**
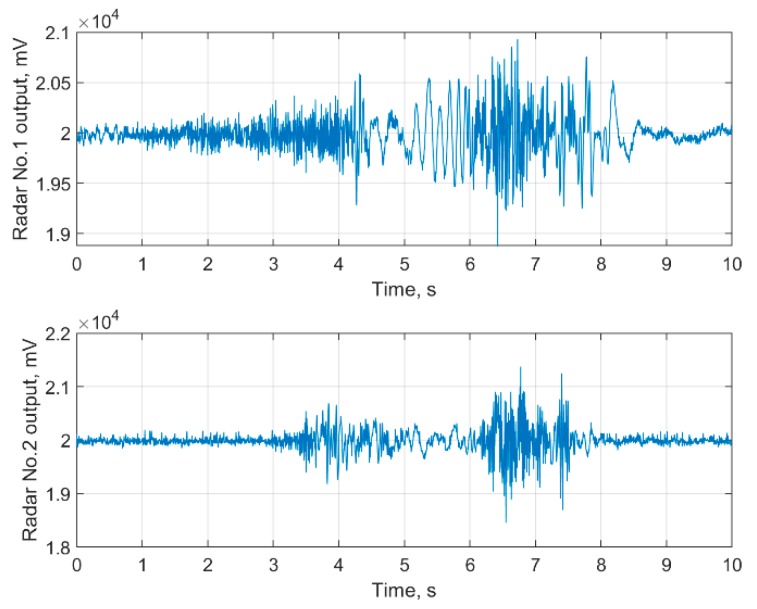
The raw bioradar signals of a human fall occurred at 6.1 s for frontal (**upper panel**) and lateral (**lower panel**) oriented bioradars.

**Figure 6:**
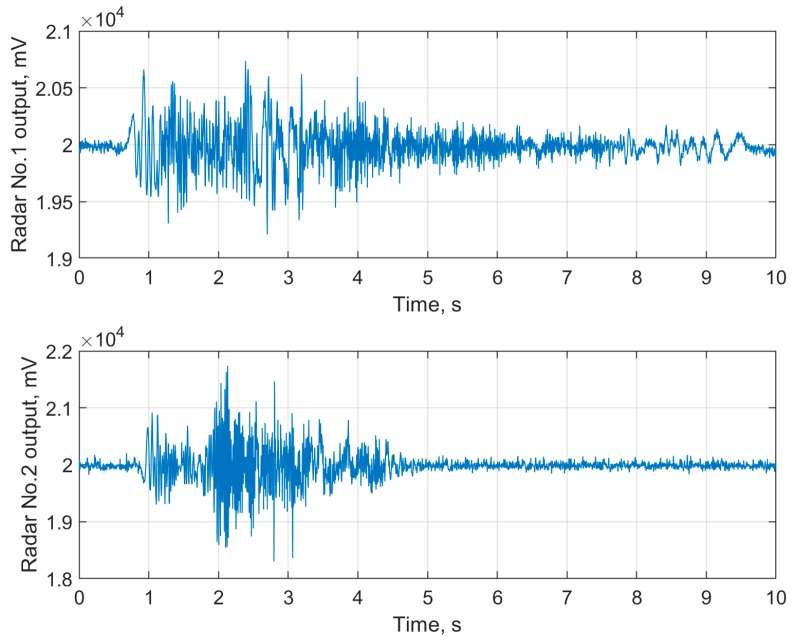
The raw bioradar signals without fall episodes for frontal (**upper panel**) and lateral (**lower panel**) oriented bioradars.

In Figure 5, examples of raw data (I quadratures) with a single fall episode registered by frontal and lateral oriented bioradars are shown. An example of raw data labeled as ‘not fall’ for the same radars is given in Figure 6. 

### 2.3. Signal Processing Technique

In the present work, we used the data processing algorithm consisting of two sequential steps: (i) preliminary data processing and (ii) learning and inference. Signal processing was done in Python 3.7 and Matlab 2019 environment.

#### 2.3.1. Preliminary Data Processing

When using radar with a quadrature receiver, there is always one channel (I or Q) that ensures the best sensitivity because of the null and optimal detection point problem [27]. There are different detection schemes such as the complex linear demodulation [27] and non-linear arc-tangent demodulation [28] which allow eliminating the null/optimal point problem in systems with quadrature demodulation. 

In the present work, we did not use an arc-tangent demodulation with a DC offset compensation technique [28] since it does not always provide good results in realistic conditions when accurate DC compensation in not possible due to clutter reflection from surrounding objects and walls and receiver imperfection. Instead, we extracted a single signal by Principal Component Analysis (PCA) used for further processing, which proved to be a reliable tool of bioaradar quadrature demodulation [29]. For each bioradar, two quadratures (I and Q) were used as an input for PCA with a single component specified as an output. The Python class sklearn.decomposition.PCA was used to perform PCA [30].

**Figure 7:**
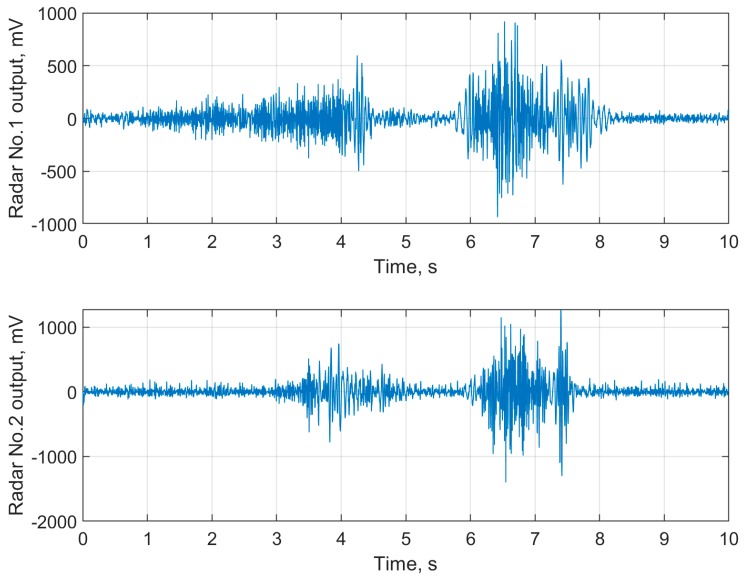
The filtered data of human fall occurred at 6.1 s for frontal (**upper panel**) and lateral (**lower panel**) oriented bioradars.

The next stage of signal processing deals with suppressing in the extracted principal component harmonics lower than 5 Hz, which may be caused by the low-frequency trend of the baseline, respiration and heartbeat of the subject as well as some everyday movement activities [31]. The cut-off frequency of 5 Hz was chosen because the fall patterns registered by radars are known to be characterized by much higher frequencies [32]. Filtration task was performed utilizing a lowpass Butterworth filter of fifth order with a cut-off frequency of 5 Hz. In Figure 7, the radar signals from Figure 4 after filtration are shown.

#### 2.3.2. Learning and Inference

In most papers dealing with fall detection problem, it is proposed to select features from the raw data, construct a feature vector with a feature extraction technique, and use this vector for training a classifier [18,22,24]. Feature selection and extraction are methods used to convert the raw data into a low-dimensional subspace that contains all relevant information for a further classification step [33]. The main problem of such an approach is that selecting features for a specific problem may be a quite challenging task, since it may require manual selection. So there is always a risk of missing relevant features, which may be crucial for successful classifier training.

**Figure 8:**
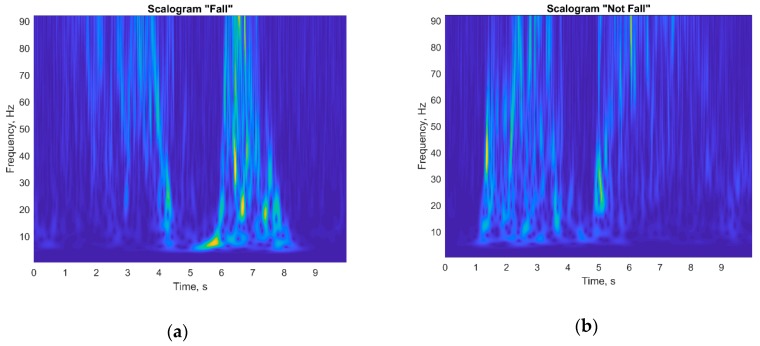
Scalograms of filtered signals for frontal-oriented bioradar: (**a**) with human fall occurring at 6.1 s; (**b**) without fall.

In this paper, it was decided not to use features selection and extraction techniques. Instead, we used the Continuous Wavelet Transform (CWT) to get a scalogram of bioradar data, which was used as an input of the pre-trained Convolutional Neuron Network (CNN). A scalogram represents the absolute values of the wavelet transform coefficients. As a base wavelet, we used an analytical Morlet (Gabor) wavelet with a number of voices per octave equal to 12. Calculating a scalogram for a filtered experimental bioradar signal required only 0.05 s while performing using Intel Core i7-920 CPU. Figure 8a,b represents scalograms of the filtered experimental signals with and without a fall episode, respectively.

Such an approach when bioradar data are transformed into a scalogram helps avoiding manual features selection and allows transfer learning of the classifier, since the useful signal information is represented as image patterns for the classification of which modern powerful pre-trained CNNs were designed.

Deep training of the CNN from scratch requires significant time and a huge amount of training data (millions of examples). This fact limits the application of the CNN in areas for which obtaining a sufficient amount of training data by experiment and synthesizing new realistic training examples are both impossible. One such area of application is fall detection. However, to deal with such a task, it is possible to adapt the CNN, designed to solve similar problems and pre-trained on a large dataset. This technique is called Transfer Learning [34].

In this work, we used the architecture of AlexNet [35], which was previously trained to recognize images of 1000 classes. This CNN is available in MATLAB by installing Neural Network Toolbox^TM^ Model for AlexNet Network. To be compatible with the AlexNet architecture, each scalogram was converted to be an array of size 227-by-227-by-3.

**Figure 9:**
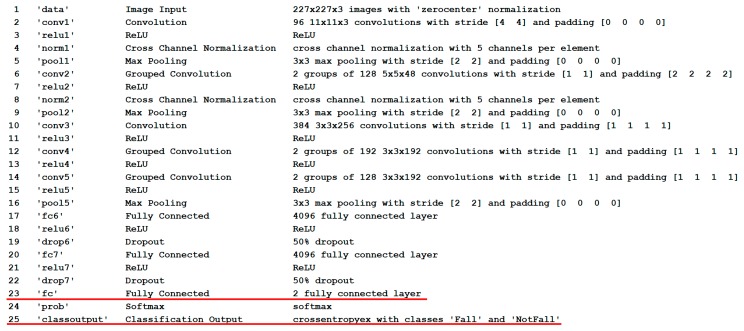
CNN architecture.

Originally, the last three layers of AlexNet were configured for recognizing 1000 categories. The following changes were made to these layers to adapt this CNN to the fall classification problem. Layer 23, the fully connected layer, was set to have the same size as the number of categories in our radar data. Layer 24 applies a softmax function to the input, so no changes are needed. The Classification Output (layer 25) holds the class labels. Since there are two bioradar data categories (‘fall’ and ‘not fall’), we set layer 23 to be a fully connected layer with two nodes, and layer 25 was set to be the classification output layer with classes ‘fall’ and ‘not fall’. The architecture of the used CNN is given in Figure 9, where red lines indicate the modified layers.

As described above, the experimental dataset contains signals from five subjects: 350 in total, 175 of which contain a fall episode. In order to train the CNN and evaluate its performance, the dataset was split taking data from first three subjects as a training set (60%, 210 records) and data for the remaining two subjects as a test set (40%, 140 records).

## 3. Results

The CNN was trained to distinguish between “fall” and “not fall” patterns. Firstly, training was carried out using the scalograms of data recorded by bioradars No. 1 and No. 2 independently. The CNNs trained on bioradar No. 1 and bioradar No. 2 data were named as CNN_1_ and CNN_2_, respectively. Their performance was estimated on two test datasets, the first of which consisted of data measured by the same bioradar as in the train dataset for the CNN, and the second dataset contained data from the other bioradar. In order to measure the performance of the proposed classifiers, we used the following metrics: accuracy, sensitivity, specificity, precision (positive predictive value), and F1-score. The classification results are listed in Table 3. It can be seen that both classifiers performed better on the dataset for the frontally-oriented bioradar (No. 1) than on the dataset for the laterally-oriented bioradar (No. 2). Moreover, the classifier performance was much better on the test dataset for the same bioradar they were trained on. Both these facts mean that a single bioradar can reliably detect falls only in cases when it has a frontal orientation toward the falling person, which makes such an approach not robust enough to be applied in real life, when positions of the falling person toward a bioradar may be different.

**Figure 10:**
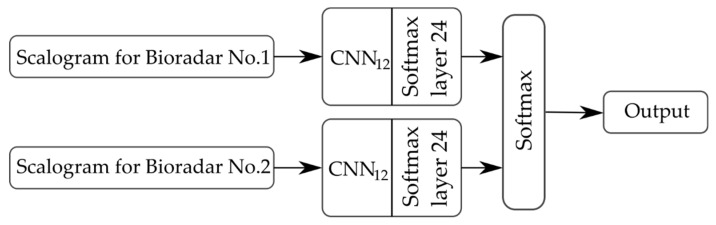
Flowchart for multi-bioradar system data classification.

To create a more robust classifier, we used data from both bioradars to train CNN (denoted by CNN_12_). In this case, the scalograms for bioradars No. 1 and No. 2 were processed by CNN_12_ separately, and the probabilities estimated by the softmax layer 24 for both scalograms were combined. The output class was picked as the class with the highest probability as is shown in Figure 10. The classification results for a multi-bioradar system are listed in Table 4.

## 4. Discussion

To the best of our knowledge, there are no open datasets for radar signals with fall patterns recorded in realistic conditions. So, there is no direct way of comparing our results with others previously achieved. However, the achieved accuracy can be indeed compared with other fall detection techniques, such as the ones provided in Table 5.

As can be seen, the accuracy of the proposed method is good compared to other reports found in the literature. Cases with the highest accuracies merge vision and depth cameras, the usage of which may rise privacy issues. On the contrary, the currently proposed solution achieved 99.29% with a non-vision method that does not violate the privacy of the person under observation, furthermore, it is much cheaper compared to the techniques based on vision or depth cameras usage. Moreover, the proposed algorithm shows the way to overcome the problem of view dependency of the radar-based fall detectors performance. The proposed classification algorithm may be applicable for a wider range of fall detection techniques: radar-based, camera and depth sensors-based.

The achieved results should be accepted with caution because the experimental dataset used for the classifier training is relatively small and acquired only for young volunteers. Nevertheless, in [42], it is shown that datasets for young people can be used instead of the one for elderly people while training a fall classifier.

The work might contribute to the development of a non-wearable and non-vision system for fall detection not only of elderly people but also for other age groups.

In the future, we are planning to enrich the experimental dataset and to extend the research by taking into account changes in environmental conditions and the presence of different types of occlusions. This will help to estimate the influence of furniture and background objects on the possibility to reliably detect falls by the proposed method. Moreover, it is planned to investigate the possibilities for optimizing the parameters of bioradars installation and to estimate the optimal number of bioradars connected into the network depending on the size of the room.

## Figures and Tables

**Table 1:** Technical Characteristics of the Bioradar.

Parameter	Bioradar No. 1	Bioradar No. 2
Probing frequency	24.107 GHz	24.065 GHz
VCO input	0 V	1.8 V
Detecting signal band	1–100 Hz	
Gain	15–30 dB	
Radiated power density	<3 µW/cm^2^	
Beam aperture	80°/34°	
Size	95 × 75 × 45 mm	

**Table 2:** Information about the studied subjects.

Male:Female	2:3
Age (Years)	22–41
Height (cm)	164–185
Body Mass Index (kg/m^2^)	17.4–22.1

**Table 3:** Experimental Dataset.

Movement Type		Number	
	Entering–exiting the premises	175$\left\{ \right.$	25
	Whole body turning		25
	Arm movements		25
Not fall activities	Sitting on the chair and standing from it		25
	leaning		25
	squats		25
	lying down on the mat		25
Falls		175	
All types of movements		350	

**Table 4:** Classification results.

CNN Name	Test Dataset	Accuracy, %	Sensitivity, %	Specificity, %	Precision, %	F1-score, %
CNN_1_	Bioradar 1	98.57	97.14	100	100	98.55
CNN_2_	Bioradar 2	87.86	85.71	90.00	89.55	87.59
CNN_2_	Bioradar 1	95.71	92.86	98.57	98.49	95.59
CNN_1_	Bioradar 2	77.14	58.57	95.71	93.18	71.93
CNN_12_	Bioradars 1&2	99.29	98.57	100	100	99.28

**Table 5:** Comparison of techniques for fall detection.

Ref.	Type of Sensors	Classifier	Amount of Channels	Number of Examinees	Accuracy, %
Martínez-Villaseñor (2019), [18]	Wearable, infrared sensors, cameras	RF, SVM, MLP, kNN	14	17	95.0
Martínez-Villaseñor (2019), [18]	cameras	CNN	2	17	95.1
Kwolek (2015), [36]	Kinect and Accelerometer	KNN and SVM	2	5	95.8
Erol (2018), [37]	Radar	STFT, GPCA and KNN	1	14	97.0
Jokanović (2017), [38]	Radar	Spectrogram and neural network	1	3	97.1
Anishchenko (2018) [39]	Camera	CNN	1	4	98.9
This work	Radars	CWT and CNN	2	5	99.3
Kwolek (2014), [40]	Kinect	KNN	1	30	100
Mastorakis (2014), [41]	Kinect	Threshold and Shape Features	1	2	100
